# The Mursa Protocol: A Novel Multimodal Antiseptic-Based DAIR Strategy for Early Hip Periprosthetic Joint Infection

**DOI:** 10.3390/antibiotics15060535

**Published:** 2026-05-25

**Authors:** Slavko Čičak, Josip Kocur, Dino Gregorović, David Matić, Dalibor Kristek, Damjan Dimnjaković, Matej Tomić, Ivan Sabol, Petra Čičak, Krunoslav Šego, Gordana Kristek, Ivana Haršanji Drenjančević

**Affiliations:** 1 Faculty of Medicine, Josip Juraj Strossmayer University of Osijek, 31000 Osijek, Croatiakristekd@yahoo.com (D.K.); plukinac@gmail.com (P.Č.); gordanaldrmed@yahoo.com (G.K.);; 2 Department of Orthopedics and Traumatology, University Hospital Centre Osijek, 31000 Osijek, Croatia; 3 Department of Orthopaedic Surgery, University Hospital Centre Zagreb, 10000 Zagreb, Croatia; 4 Department of Pulmonology and Intensive Care Medicine, University Hospital Centre Osijek, 31000 Osijek, Croatia; 5 Department of Cardiac and Thoracic Surgery, University Hospital Centre Osijek, 31000 Osijek, Croatia; 6 Department of Anesthesiology, Resuscitation and ICU, University Hospital Centre Osijek, 31000 Osijek, Croatia

**Keywords:** arthroplasty, prosthesis-related infections, anti-bacterial agents, debridement, treatment outcome

## Abstract

**Background:** Debridement, antibiotics, and implant retention (DAIR) is an established treatment for early periprosthetic joint infection (PJI) following hip arthroplasty; however, reported success rates remain highly variable, particularly in patients with significant comorbidities, fracture-related arthroplasty, or resistant microorganisms. Augmentation of standard DAIR with structured local antimicrobial strategies may improve infection control but remains insufficiently standardized and evaluated. **Methods:** This retrospective single-center case series evaluated outcomes of a standardized multimodal DAIR-based strategy, the Mursa protocol, in 16 consecutive patients treated for early hip PJI between 2022 and 2025. PJI was diagnosed according to European Bone and Joint Infection Society criteria. The treatment included radical surgical debridement and exchange of mobile components with sequential intraoperative antiseptic microdebridement using povidone–iodine and hypochlorous/hypochlorite solution, followed by postoperative drain-based local antimicrobial irrigation and systemic antibiotic therapy. Treatment success was defined as sustained infection eradication with implant retention, absence of clinical and radiological signs of infection, no requirement for long-term suppressive antibiotics, and no infection-related mortality at a minimum one-year follow-up. **Results:** The cohort was clinically complex, with a predominance of arthroplasty procedures performed for fracture-related indications (11/16), a high comorbidity burden (median Charlson Comorbidity Index 5), revision arthroplasty in four patients, and a high rate of resistant or polymicrobial infections. At final follow-up, 15 of 16 patients (93.8%) achieved treatment success. One patient required implant removal due to persistent polymicrobial infection. No irrigation-related complications, wound-healing problems, or clinically relevant systemic toxicity were observed. **Conclusions:** In this high-risk cohort, a structured multimodal DAIR protocol incorporating sequential antiseptic microdebridement and postoperative local antimicrobial irrigation was feasible, safe, and associated with encouraging infection control. However, these findings should be interpreted as hypothesis-generating, and further prospective comparative studies are required to validate the protocol.

## 1. Introduction

Treatment of periprosthetic joint infection (PJI) remains a significant therapeutic challenge but also a major financial and public health burden. Effective management requires a coordinated combination of surgical intervention and antimicrobial therapy [1,2,3]. While two-stage revision continues to be regarded as the gold standard for chronic PJI, debridement, antibiotics, and implant retention (DAIR) has gained increasing acceptance in carefully selected patients with early infection [4].

Most authors define early PJI as an infection occurring within 4 weeks of the index arthroplasty, with a symptom duration not exceeding 3 weeks, a time window in which patients are generally considered suitable candidates for DAIR [1,3,5]. The primary goal of DAIR as a curative procedure is the eradication of infection while retaining the prosthesis. The major obstacle to successful treatment is bacterial biofilm formation, which begins shortly after bacterial adherence to the implant surface and matures over time. When successful, DAIR offers several advantages, including reduced morbidity, improved functional outcomes, and lower healthcare costs compared with revision strategies [6].

Although debridement with implant retention was described in earlier literature [7], the concept of curative DAIR as a structured treatment algorithm—involving a single surgical intervention with exchange of mobile components followed by prolonged systemic antibiotic therapy (3–6 months)—was first clearly defined by a specialized Swiss PJI group [3]. Since then, numerous modifications of the DAIR procedure have been proposed, with highly variable outcomes. Reported success rates across published studies range from 11% to 100% in a total of 4987 PJI cases, underscoring the substantial heterogeneity of outcomes in the literature [8].

Several factors contribute to this wide variability in outcomes. Successful DAIR has been associated with pathogen-related factors, including virulence, antimicrobial resistance, and biofilm-forming capacity; host-related factors, such as immune status and comorbidities; surgical factors, particularly the quality and radicality of debridement; and antimicrobial factors, including appropriate pathogen-directed therapy of sufficient dose and duration. When these factors are optimally addressed, success rates exceeding 90% have been reported in primary total hip arthroplasty (THA), comparable to outcomes achieved with two-stage revision for chronic PJI. This highlights the importance of early infection detection and appropriate patient selection for DAIR [5,9,10,11,12].

In contrast, success rates are significantly lower in hip fracture patients with substantial comorbidity, ranging from 22% to 53% [2,13,14]. Additional predictors of poor outcome include infection with multidrug-resistant organisms, polymicrobial infections, and fungal PJI, which are often considered relative contraindications for DAIR [6].

To address these limitations, several modifications of the classical DAIR approach have been explored. Intraoperative use of local antibiotic and antiseptic solutions has been associated with improved outcomes in selected studies; however, these strategies remain heterogeneous and incompletely standardized [12,15,16,17,18]. Moreover, while postoperative local antibiotic infusion has been described, data regarding the efficacy, safety, and reproducibility of postoperative local antiseptic irrigation in early PJI are scarce, and its role within a structured DAIR algorithm remains poorly defined [19,20,21,22,23].

In this study, we describe the Mursa protocol, a standardized perioperative DAIR-based strategy for early hip PJI that integrates radical debridement, sequential intraoperative antiseptic microdebridement, and postoperative drain-based local antimicrobial therapy in combination with systemic antibiotics. We report the clinical outcomes of this protocol and aim to evaluate its feasibility and infection control potential in a consecutive series of patients.

## 2. Materials and Methods

This study is a retrospective, single-center observational case series analyzing the outcomes of a standardized DAIR-based treatment protocol in 16 consecutive patients treated with the Mursa protocol between 2022 and 2025. The Mursa protocol represents a structured institutional treatment approach based on established surgical and antimicrobial principles routinely used in clinical practice and does not constitute an experimental intervention. Periprosthetic joint infection was diagnosed according to the modified criteria of the European Bone and Joint Infection Society (EBJIS, 2021) [1].

The study was conducted in accordance with institutional review board requirements for retrospective observational research. Inclusion criteria comprised all patients who consented to treatment for early periprosthetic infection following primary partial hip arthroplasty, primary total hip arthroplasty, or revision total hip arthroplasty. Additional inclusion requirements were the presence of a stable implant—defined by the absence of radiographic signs of loosening and confirmed intraoperatively—and adequate soft-tissue conditions at the time of surgery.

**Figure 1:**
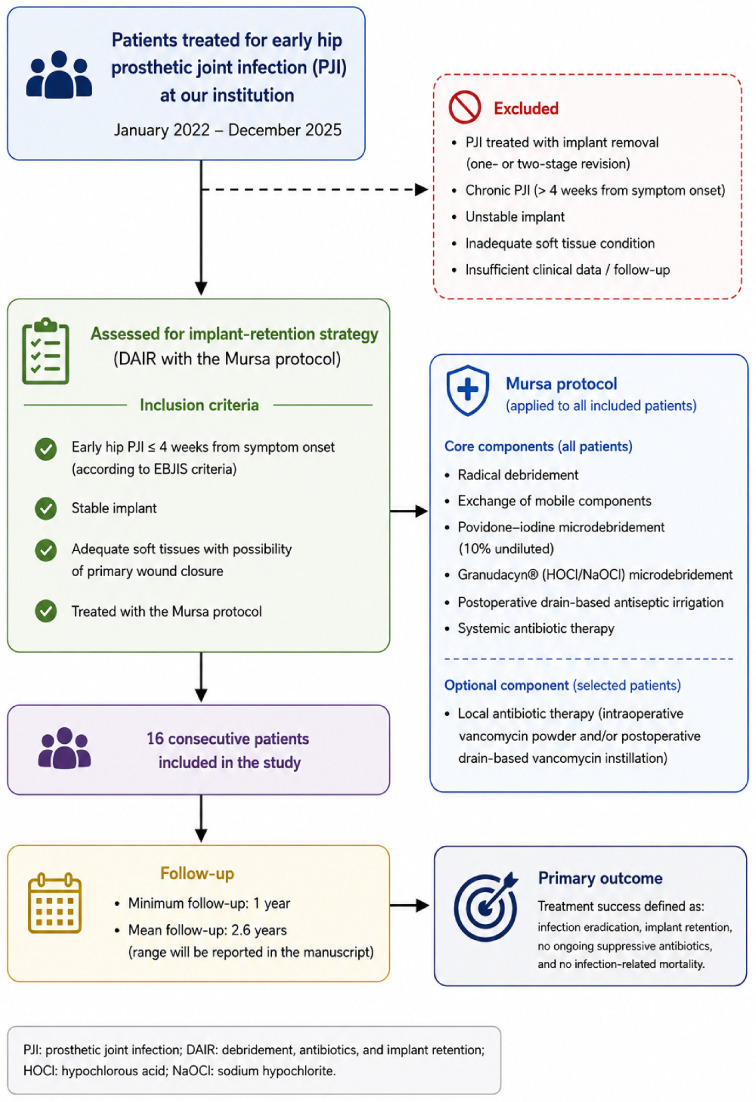
Simplified study schematic illustrating patient inclusion criteria.

All infections included in this study occurred in patients primarily operated at our institution; however, a subset of cases involved revision arthroplasty procedures that had been referred from other centers due to their complexity. The study cohort represents a consecutive series of patients treated with the Mursa protocol by the surgical team that developed it. It does not include all patients with periprosthetic joint infection treated at our institution during the study period. To improve transparency of cohort selection, a simplified study schematic illustrating patient inclusion criteria and selection of consecutive patients treated with the Mursa protocol has been added (Figure 1).

Patient follow-up was conducted from the day of surgery until the most recent clinical evaluation or until recurrence of infection, at which point the DAIR protocol was considered unsuccessful. The minimum follow-up was one year. Treatment failure was defined by the occurrence of any of the following: requirement for additional surgical intervention due to infection after DAIR; presence of a persistent sinus tract, ongoing wound drainage, or excessive joint pain attributable to infection; need for long-term suppressive antibiotic therapy; relapse of infection caused by the same pathogen; reinfection with a different microorganism; or infection-related death.

Treatment success was defined as eradication of infection with retention of the implant, characterized by the absence of clinical signs of infection (including sinus tract formation, wound drainage, or infection-related pain), no requirement for additional surgical intervention, no need for long-term suppressive antibiotic therapy, no microbiological evidence of relapse or reinfection, and normalization of inflammatory laboratory markers (particularly CRP) consistent with clinical recovery.

Follow-up was conducted according to a structured protocol. During hospitalization, the surgical wound was assessed daily. Laboratory evaluations were performed on postoperative days 5, 8, and 15, in addition to immediate postoperative blood testing. These included assessment of inflammatory markers, liver and renal function parameters, and total protein levels.

After discharge, follow-up visits were scheduled at approximately 2 weeks, 1 month, 3 months, 6 months, and 12 months postoperatively, and annually thereafter. Each visit included clinical and laboratory assessment.

The projected DAIR success rate was calculated for each case using the modified KLICC (acronym for Kidney, Liver, Index surgery, Cemented, C-reactive protein) score. Additionally, patient complexity was analyzed using the Charlson Comorbidity Index and the Comorbidity–Polypharmacy Score. All patients provided written informed consent for surgical treatment, including the use of local antimicrobial and antiseptic measures as part of infection management.

### 2.1. Mursa Protocol Description

The Mursa protocol (named after the ancient Roman name for Osijek and not used as an acronym) is divided into three phases: preoperative preparation, surgical intervention combined with local antimicrobial irrigation (DAIR with combined antiseptic and antibiotic treatment), and postoperative antimicrobial irrigation combined with systemic antibiotic therapy.

#### 2.1.1. Preoperative Preparation

Periprosthetic hip joint infection (PJI) was confirmed using the EBJIS definition in patients with clinical findings suggestive of infection, including persistent wound drainage, erythema, swelling, local warmth, pain, delayed wound healing, and, in some cases, systemic signs such as fever [1]. Surgical management was scheduled as early as feasible. To maximize culture yield, no antibiotics were administered preoperatively. If the patient was receiving antibiotics at presentation, antimicrobial therapy was withheld until after surgical debridement and collection of intraoperative specimens (unless the patient was septic or clinically unstable) [24].

Preoperative laboratory evaluation included inflammatory markers (CRP, leukocyte count, erythrocyte sedimentation rate [ESR]), renal and hepatic function, serum glucose, and nutritional parameters (including total protein and albumin). Oral protein supplementation (Abound^®^, Abbott Nutrition, Columbus, OH, USA) was administered as a supportive measure in the perioperative period, particularly in the context of increased metabolic demand associated with infection and surgical treatment. Laboratory parameters, including total protein and albumin levels, were assessed preoperatively and monitored during hospitalization and follow-up; however, a standardized analysis of changes before and after supplementation was not systematically performed.

#### 2.1.2. Operative Treatment—DAIR Procedure + Local Antimicrobial Irrigation

The operative technique had two principal aims: to accurately identify the causative organism(s) in order to optimize postoperative antibiotic therapy, and to eradicate the infection. The hip was approached through the previous incision. The prior scar was not routinely excised; however, necrotic skin edges and any nonviable wound tissue were sharply debrided when present. Preservation of viable tissue was guided by intraoperative assessment of tissue viability based on established surgical criteria, including tissue color, consistency, presence of punctate bleeding, and response to irrigation.

Microbiological sampling: Multiple deep periprosthetic tissue samples were collected during dissection in a layer-by-layer manner (subcutaneous, subfascial, and deep/periarticular), including samples obtained from beneath mobile components. A minimum of five tissue samples was obtained, with additional specimens collected when loculated tissue planes or fluid collections were encountered. Swab samples were not routinely used.

**Debridement and Component Exchange:** All macroscopically necrotic and avital tissue was excised (“macrodebridement”) from superficial to deep planes using a scalpel and curettes or rongeurs as required. Throughout macrodebridement, the wound was repeatedly irrigated with 2–4 L of sterile saline, adjusted according to the extent of soft-tissue involvement. The femoral head and polyethylene liner were removed to reduce bioburden, improve access to implant surfaces, and enable more complete debridement. Implant stability was assessed at this stage. Structured Antiseptic Microdebridement: As an augmented component of our DAIR protocol, a stepwise antiseptic microdebridement protocol was then performed to further reduce the microbial load using povidone–iodine followed by a hypochlorous/hypochlorite solution (Granudacyn^®^, Mölnlycke, Gothenburg, Sweden). Between antiseptics, the wound was thoroughly irrigated with saline to minimize chemical interactions and residual carryover.

**Povidone–Iodine Step:** Undiluted 10% povidone–iodine was applied to wound edges, infected tissue, and exposed implant surfaces using a microporous sponge held with forceps. During this step, the wound was intermittently irrigated with saline every 1–2 min to remove loosened debris and dilute the antiseptic. Additional iodine-soaked gauze swabs were used for targeted microdebridement circumferentially around the femoral stem, the stem–bone interface, and the acetabular surfaces. We are aware of the potential cytotoxic effects of povidone–iodine, particularly at higher concentrations. In the present protocol, however, undiluted 10% povidone–iodine was intentionally used as part of a brief, mechanically assisted “microdebridement” strategy rather than as a prolonged lavage solution. The rationale was to achieve localized chemical and mechanical reduction in microbial burden on contaminated tissue surfaces and implant interfaces while potentially limiting the extent of radical macrodebridement. In our clinical experience, this approach may allow preservation of greater amounts of viable soft tissue compared with more extensive excisional debridement, although this concept remains theoretical and was not specifically evaluated in the present study. To minimize prolonged tissue exposure and residual antiseptic burden, repeated saline irrigation was performed throughout this phase of the procedure.

Tissue viability was assessed intraoperatively based on established surgical criteria, including tissue color, consistency, and the presence of capillary (punctate) bleeding, which are commonly used indicators of viable tissue during debridement [25,26].

In addition, we subjectively observed that some viable tissues appeared to lose antiseptic staining following irrigation, whereas persistently stained areas were reassessed intraoperatively and, if considered suspicious for nonviability based on conventional surgical criteria, were further debrided. This observation reflects intraoperative experience and should be interpreted as an adjunctive finding rather than a validated criterion.

An additional 2–4 L of saline was used during this phase, and irrigation was continued until visible antiseptic staining was no longer present.

**Granudacyn Step:** Wound edges and visible implant surfaces were then wiped multiple times with Granudacyn-soaked gauze. The wound was filled to the level of the skin edges and left in situ for 1–2 min, in accordance with the manufacturer’s instructions.

New modular components were implanted, hip stability was reassessed, and increased constraint was used when necessary, without revising the fixed femoral or acetabular components.

Optional topical antibiotic therapy (vancomycin powder) was used selectively in cases with suspected or confirmed Gram-positive infection, particularly when organisms were expected to be vancomycin-susceptible. This was not a routine component of the protocol and was applied at the discretion of the surgical team. The rationale for local antibiotic application was to achieve high local antimicrobial concentrations immediately after debridement. This was followed by postoperative antiseptic irrigation, which was intended to act in a complementary manner by further reducing microbial burden during the early postoperative period.

**Figure 2:**
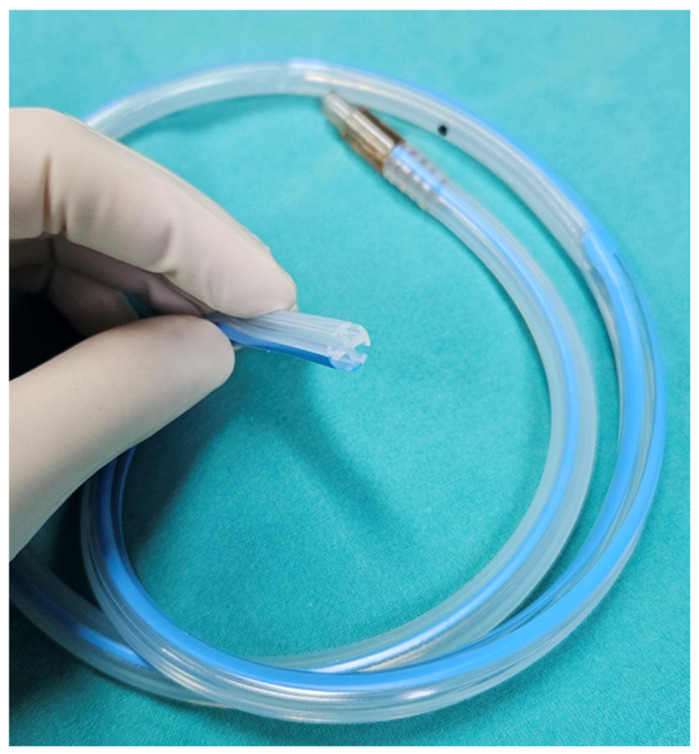
View of the Blake drain used for postoperative irrigation and drainage of the operated hip.

**Drain Placement and Closure:** A single intra-articular drain (Blake 24 Fr silicone drain (Ethicon, Somerville, NJ, USA); Figure 2) was placed to facilitate postoperative irrigation and to reduce the risk of clogging compared with conventional drains. The drain was placed intra-articularly under direct visualization at the end of the procedure and exited the skin at a distance from the primary incision to ensure an adequate soft-tissue bridge and facilitate independent dressing. Owing to the use of microdebridement techniques enabling tissue-sparing debridement, watertight fascial closure was achieved, maintaining a contained subfascial space and allowing controlled pressurization during irrigation. Although the precise distribution of the irrigation fluid cannot be directly verified, instillation was performed in a controlled manner to preferentially target this space while minimizing soft-tissue extravasation.

#### 2.1.3. Postoperative Antimicrobial Irrigation Combined with Systemic Antibiotic Therapy

Parenteral antibiotic therapy was initiated immediately after surgery and was continued for 14 days in accordance with the PRO-IMPLANT Foundation protocol for periprosthetic joint infection [24]. Antibiotic selection was empirical until definitive culture results became available, at which point the regimen was adjusted based on pathogen identification and susceptibility testing.

**Drain-Based Irrigation** (Postoperative Days 1–7); For the first 7 postoperative days following DAIR, the joint cavity was irrigated once daily via the drain. During each irrigation session, the instillation–aspiration cycle using antiseptic solution was repeated three times according to our institutional protocol. The concept of local postoperative antimicrobial delivery in periprosthetic joint infection has been described in the literature using various intra-articular and catheter-based systems [11,12,17,27,28]. In the present study, the postoperative irrigation technique represents an institutional adaptation of these principles. Although the exact drain configuration and irrigation protocol applied in this study have not been independently validated in prior literature, they were implemented under controlled conditions as part of a structured clinical treatment approach. While no irrigation-related complications were observed in this cohort, the safety and effectiveness of this specific postoperative irrigation strategy require further prospective evaluation.

The suction reservoir was disconnected, and the drain was handled under strict aseptic conditions, including cleaning and disinfection, to minimize the risk of contamination during irrigation. Residual intra-articular fluid was aspirated via the drain using a sterile syringe, and the cavity was then gently pressurized with 30–50 mL of Granudacyn (adjusted to the estimated dead space). The infusion was stopped if the patient experienced unacceptable wound tension or pain. The drain was clamped for 1–2 min, after which the fluid was aspirated.

**Figure 3:**
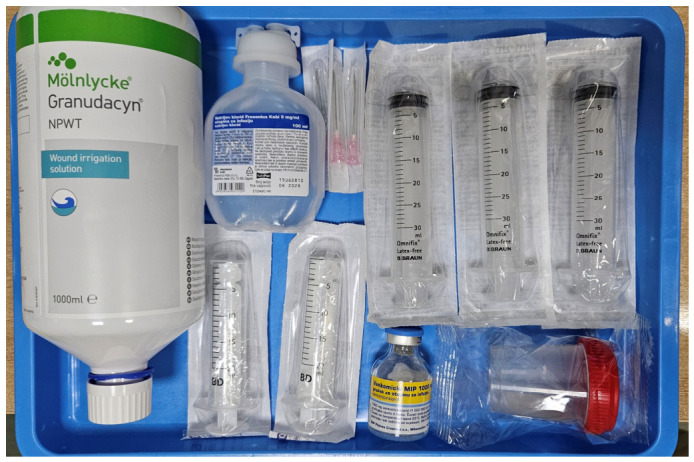
Presentation of the materials required for daily irrigation of the hip joint via a Blake drain. The image shows Granudacyn solution, three 30 mL syringes for administering Granudacyn previously drawn from a sterile container, and a 20 mL syringe in which the antibiotic (vancomycin) is dissolved and then locally administered through the drain.

In selected cases of low-grade infection caused by vancomycin-susceptible organisms, a vancomycin solution (powder reconstituted in normal saline) was instilled via the drain and temporarily retained by clamping (4–6 h), according to our institutional protocol. The materials used for this are shown in Figure 3.

**Drain Removal and Wound Course**: On postoperative day 8, the drain was removed. Serous drainage from the drain site sometimes persisted due to local antiseptic exposure and typically decreased over 3–7 days until closure occurred.

The core components of the Mursa protocol—radical debridement, exchange of mobile components, intraoperative antiseptic microdebridement, postoperative drain-based antiseptic irrigation, and systemic antibiotic therapy—were applied in all patients. In contrast, local antibiotic therapy (including intraoperative vancomycin powder and postoperative drain-based antibiotic instillation) was used selectively, based on suspected or confirmed pathogen susceptibility and clinical judgment.

### 2.2. Monitoring and Rehabilitation

On postoperative day 8, laboratory testing was repeated (complete blood count [CBC], C-reactive protein [CRP], urea and creatinine during parenteral therapy, and total protein levels). During the drain period, rehabilitation was continued with range-of-motion exercises. Patients were seated and progressively mobilized to an upright position (verticalized) as tolerated with crutches.

Parenteral antibiotics were continued to complete the 14-day course. On postoperative day 12, conversion planning to oral therapy was initiated based on the isolated pathogen and its susceptibility profile, consistent with PRO-IMPLANT guidance [29].

If the wound was dry by postoperative day 15, the staples were removed, and the patient was discharged with a planned 10-week course of oral antibiotics in accordance with PJI guidelines. The first follow-up visit was scheduled approximately 1 month after discharge, provided recovery was uncomplicated.

Laboratory parameters, including C-reactive protein (CRP), complete blood count, and renal and hepatic function, were routinely monitored during hospitalization and follow-up, even in the absence of clinical signs of infection, and were interpreted in conjunction with clinical findings. Follow-up assessments were performed periodically during oral antibiotic therapy, with particular attention to potential adverse effects, including those associated with prolonged fluoroquinolone use (e.g., tendinopathy and musculoskeletal symptoms). No clinically significant antibiotic-related complications requiring discontinuation of therapy were observed.

## 3. Results

### 3.1. Patient Characteristics

Sixteen patients were treated according to the Mursa protocol during the study period. The cohort consisted of 10 women and 6 men, with a mean age of 66.1 years (range, 37–91 years). Most patients were classified as ASA III (13/16), indicating a substantial burden of systemic comorbidity. The median Charlson Comorbidity Index (CCI) was 5 (range, 0–10), and the median comorbidity–polypharmacy score (CPS) was 10.5 (range, 0–25), shown in Table 1.

The indication for arthroplasty was fracture-related in the majority of cases, including femoral neck fractures, periprosthetic fractures and failed intertrochanteric fracture treatment. Four patients underwent revision total hip arthroplasty: two due to femoral periprosthetic fractures with unstable stems, one after failed intertrochanteric fracture osteosynthesis, and one for aseptic acetabular loosening following primary total hip arthroplasty. Only one patient was operated on for primary osteoarthritis of the hip joint. Implant types included primary total hip arthroplasty (THA) in 11 patients, revision THA in 4 patients, and cemented hemiarthroplasty in 1 patient.

### 3.2. Infection Characteristics

All cases fulfilled the criteria for early periprosthetic joint infection. The median onset of symptoms occurred 9.5 days after index surgery (range, 5–29 days). The median time from symptom onset to DAIR was 5 days (range, 3–22 days).

The median modified KLICC score was 5.75 (range, 3–9), indicating a moderate predicted risk of DAIR failure in this cohort.

A causative microorganism was identified in 15 of 16 cases. Coagulase-negative staphylococci were the most frequently isolated pathogens, predominantly Staphylococcus epidermidis (11/16), including methicillin-resistant strains. Other identified organisms included Enterococcus faecalis, Staphylococcus aureus, Acinetobacter baumannii, Klebsiella aerogenes, Enterobacter cloacae, and polymicrobial infections. One case was culture-negative.

### 3.3. Treatment Details

All patients underwent DAIR with exchange of mobile components and application of the standardized intraoperative and postoperative antimicrobial protocol. Parenteral antibiotic therapy was administered for 14 days in all cases, followed by 10 weeks of oral antibiotic therapy, tailored according to culture results and antimicrobial susceptibility testing. Vancomycin-based regimens, combined with rifampicin or beta-lactams, were most commonly used in staphylococcal infections.

Postoperatively, drain-based local antiseptic irrigation and protocol-defined local antibiotic irrigation were performed in all patients according to the Mursa protocol. Optional local antibiotic therapy (intraoperative vancomycin powder and/or postoperative drain-based vancomycin instillation) was used selectively in 6 of 16 patients. No irrigation-related adverse effects were observed, and no patient developed clinically relevant wound complications, soft-tissue reactions, or renal impairment attributable to local antiseptic or antibiotic irrigation.

### 3.4. Clinical Outcomes

At final follow-up, 15 of 16 patients (93.8%) met the predefined criteria for treatment success, with eradication of infection and retention of the implant. The mean follow-up duration was 2.6 years (range, 1–4.1 years). These patients remained symptom-free, without evidence of recurrent infection, need for suppressive antibiotic therapy, or infection-related mortality. One patient (6.2%) experienced treatment failure, requiring implant removal due to persistent infection. This case involved a polymicrobial infection with methicillin-resistant coagulase-negative staphylococci and Klebsiella aerogenes following primary THA for femoral neck fracture.

No infection-related deaths were observed. No cases of persistent sinus tract formation, prolonged wound drainage, irrigation-related complications, or antibiotic-related adverse events requiring discontinuation of therapy were recorded during follow-up. Clinical characteristics and outcomes of the study cohort are shown in Table 2.

## 4. Discussion

### 4.1. Principal Findings

**Figure 4:**
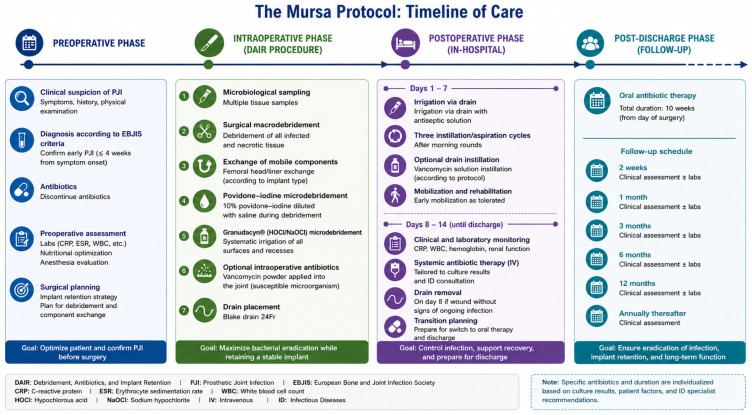
The Mursa Protocol: Timeline of Care.

The present study evaluated the feasibility, safety, and clinical outcomes of a standardized, multimodal DAIR-based treatment strategy—the Mursa protocol—for the management of early periprosthetic joint infection of the hip. In this consecutive case series of 16 patients, treatment using this protocol was associated with a success rate of 93.8%, following the structured timeline of care illustrated in Figure 4, defined as infection eradication with implant retention at final follow-up.

Importantly, these results were obtained in a cohort characterized by high clinical complexity, including a predominance of arthroplasty procedures performed for fracture-related indications, a substantial burden of comorbidities, frequent use of revision implants, and the presence of resistant and polymicrobial infections. Despite these recognized risk factors for DAIR failure, the protocol was successfully implemented in all patients and was not associated with irrigation-related adverse events, wound complications, or clinically relevant systemic toxicity.

From a clinical perspective, these findings should be interpreted as hypothesis-generating. However, they suggest that a structured integration of radical debridement, sequential intraoperative antiseptic microdebridement, and postoperative local antimicrobial irrigation, combined with targeted systemic antibiotic therapy, is feasible and appears safe in selected patients with early hip PJI. While the present study is based on a small cohort of patients and is therefore not designed to demonstrate superiority over standard DAIR, the observed infection control rate supports further investigation of this multimodal approach, particularly in patient populations traditionally considered at high risk for implant-retention strategies. These findings should be interpreted cautiously, given the retrospective design, small sample size, and lack of a comparator group.

**Table 2:** Clinical Characteristics and Outcomes of the Study Cohort.

	Age/Sex	ASA Classification *	Operative Indication	Implant	Onset of Symptoms (Days After Operation)	Time from Symptom Onset to DAIR (Days)	Modified KLICC Score **	Charlson Comorbidity Index (CCI) ***	Comorbidity–Polypharmacy Score (CPS)	Isolated Bacteria	Antibiotics	Outcome
1.	75 F	ASA III	Femoral neck fracture	Primary THA	5	3	4	4	8	/	Vancomycin 2 × 1 g + Ampicillin/sulbactam 3 × 3 g 2 weeks i.v. Amoxicillin 3 × 1 g + Ciprofloxacin 2 × 750 mg 10 weeks oral	Infection resolved,
2.	83 F	ASA III	Aseptic acetabular loosening after THA	Revision THA	9	7	9	9	10	*S. epidermidis*	Vancomycin 2 × 1 g i.v.+ Rifampicin 3 × 300 mg oral 2 weeks. Doxycyclin 2 × 100 mg + Rifampicin 3 × 300 mg 10 weeks oral	Infection resolved
3.	61 F	ASA III	Femoral neck fracture	Primary THA	22	14	9	10	25	*E. faecalis*	Piperacillin/tazobactam 3 × 4.5 g + Gentamicin 2 × 120 mg 2 weeks i.v. Amoxicillin 3 × 1 g + Doxycycline 2 × 100 mg 10 weeks oral	Infection resolved
4.	68 M	ASA III	Femoral neck fracture	Primary THA	5	4	6	8	12	MR-CoNS ****/*Klebsiella aerogenes*	Piperacillin/tazobactam 3 × 4.5 g 2 weeks i.v.	Failed DAIR, prosthesis extracted
5.	73 F	ASA II	Failed intertrochanteric fracture osteosynthesis	Primary THA	9	5	4	4	5	*S. epidermidis* (MRSE) *****	Vancomycin 2 × 1 g i.v.+ Rifampicin 3 × 300 mg oral 2 weeks. Doxycyclin 2 × 100 mg + Rifampicin 3 × 300 mg 10 weeks oral	Infection resolved
6.	64 M	ASA III	Periprosthetic fracture—Vancouver B2	Revision THA	21	3	6.5	6	8	*S. aureus*	Fosfomycin 3 × 5 g + Cefazolin 3 × 2 g 2 weeks i.v. Doxycyclin 2 × 100 mg + Rifampicin 3 × 300 mg 10 weeks oral	Infection resolved
7.	44 M	ASA III	Secondary posttraumatic OA after acetabular fracture ORIF	Primary THA	21	5	4	2	4	*S. epidermidis*	Vancomycin 2 × 1 g i.v.+ Rifampicin 3 × 300 mg oral 2 weeks. Doxycyclin2 × 100 mg + Rifampicin 3 × 300 mg 10 weeks oral	Infection resolved
8.	70 F	ASA III	Failed intertrochanteric fracture osteosynthesis	Revision THA	6	3	5.5	6	19	*A. Baumannii*	Fosfomycin 3 × 5 g 2 weeks i.v. Ciprofloxacin 2 × 750 mg 10 weeks oral	Infection resolved
9.	77 F	ASA III	Periprosthetic fracture—Vancouver B2	Revision THA	11	4	6.5	9	17	*S. epidermidis* (MRSE)	Vancomycin 2 × 1 g i.v.+ Rifampicin 3 × 300 mg oral 2 weeks. Doxycyclin2 × 100 mg + Rifampicin 3 × 300 mg 10 weeks oral	Infection resolved
10.	61 F	ASA III	Femoral neck fracture	Primary THA	18	7	7	6	16	*E. cloacae*/*S. caprae*	Fosfomycin 3 × 5 g + Vancomycin 2 × 1 g 2 weeks i.v. Cotrimoxazole 3 × 960 mg + Rifampicin 3 × 300 mg 10 weeks oral	Infection resolved
11.	50 M	ASA II	Hip Osteoarthritis	Primary THA	5	11	3.5	1	0	*S. epidermidis*	Flucloxacillin 4 × 2 g 2 weeks i.v. Doxycyclin2 × 100 mg + Rifampicin 3 × 300 mg 10 weeks oral	Infection resolved
12.	37 M	ASA II	Femoral head AVN after acetabular ORIF	Primary THA	15	3	4.5	0	0	*S. epidermidis* (MR-CoNS)	Vancomycin 2 × 1 g i.v.+ Rifampicin 3 × 300 mg oral 2 weeks. Levofloxacin 2 × 500 mg 10 weeks oral	Infection resolved
13.	67 F	ASA III	Femoral neck fracture	Primary THA	10	4	5	4	25	*S. epidermidis* (MRSE)	Vancomycin 2 × 1 g i.v.+ Rifampicin 3 × 300 mg oral. 2 weeks. Doxycyclin2 × 100 mg + Rifampicin 3 × 300 mg 10 weeks oral	Infection resolved
14.	78 M	ASA III	Femoral neck fracture	Primary THA	8	22	4.5	4	10	*S. epidermidis*	Vancomycin 2 × 1 g i.v.+ Rifampicin 3 × 300 mg oral 2 weeks. Doxycyclin2 × 100 mg + Rifampicin 3 × 300 mg 10 weeks oral	Infection resolved
15.	58 F	ASA III	Femoral neck fracture	Primary THA	29	7	7	4	11	*S. epidermidis*	Vancomycin 2 × 1 g i.v.+ Rifampicin 3 × 300 mg oral 2 weeks. Doxycyclin2 × 100 mg + Rifampicin 3 × 300 mg 10 weeks oral	Infection resolved
16.	91 M	ASA III	Femoral neck fracture	Cemented hemiarthroplasty	6	5	6	10	13	*S. epidermidis* (MR-CoNS)	Vancomycin 2 × 1 g i.v.+ Rifampicin 3 × 300 mg oral 2 weeks. Doxycyclin2 × 100 mg + Rifampicin 3 × 300 mg 10 weeks oral	Infection resolved

* ASA classification—American Society of Anesthesiologists Physical Status that categorizes a patient’s overall health before surgery. ** Modified KLICC score—acronym for **K**idney, **L**iver issues, **I**ndex surgery type, **C**emented prosthesis, and high **C**RP levels—a clinical tool used in orthopedics to predict the risk of treatment failure for prosthetic joint infections. *** Charlson Comorbidity Index (CCI)—a clinical tool used to categorize comorbidities and predict the risk of mortality. **** Methicillin-resistant coagulase-negative staphylococci. ***** Methicillin-resistant Staphylococcus epidermidis.

### 4.2. Comparison with Existing Literature

Reported outcomes of DAIR for early periprosthetic joint infection vary widely across the literature, reflecting substantial heterogeneity in patient selection, infection characteristics, surgical technique, and antimicrobial strategies [5,8,10,29]. Large systematic reviews and registry-based studies have reported success rates ranging from approximately 40% to 80% for hip PJI, with particularly poor outcomes observed in fracture-related cases, revision arthroplasty, and patients with significant comorbidity burden [4,5,25,26].

Several studies have identified fracture-related arthroplasty, advanced age, high comorbidity indices, and revision implants as independent predictors of DAIR failure [5,6,21,29]. In hip fracture populations, reported success rates are consistently lower, often ranging between 20% and 50%, even when DAIR is performed within recommended time windows [2,14]. Similarly, elevated KLIC or Charlson Comorbidity Index scores have been associated with markedly reduced implant retention rates following DAIR, with reported revision-free survival at 24 months ranging from 42% to 76% [30,31].

Microbiological factors further contribute to outcome variability. Infections caused by methicillin-resistant staphylococci, polymicrobial organisms, and Gram-negative pathogens have been repeatedly associated with inferior results compared with infections caused by methicillin-susceptible Staphylococcus aureus. As a consequence, several authors consider resistant or polymicrobial infections to represent relative contraindications to implant retention strategies [6,26,30].

Against this background, the infection control rate observed in the present study appears favorable, particularly given the high-risk profile of the treated cohort.

With respect to surgical technique, previous studies have emphasized the importance of radical debridement, exchange of mobile components, and pathogen-directed systemic antibiotic therapy as core elements of successful DAIR [5,26]. More recent investigations have explored the adjunctive use of local antimicrobial strategies, including intraoperative antiseptic or antibiotic lavage, with some reporting improved infection control. However, these approaches remain inconsistently applied, and evidence regarding postoperative local antiseptic irrigation is limited [15,20,21,23,31,32].

The present findings add to the growing body of literature suggesting that structured augmentation of standard DAIR, including sequential antiseptic microdebridement and controlled postoperative local antimicrobial irrigation, may potentially contribute to infection control in selected patients. While direct comparison with existing series is limited by differences in study design and patient populations, the results may support further investigation in cohorts traditionally considered at high risk for failure.

### 4.3. Role of Local Antimicrobial Agents

Eradication of biofilm-embedded microorganism adherent to an avital prosthetic surface by the host immune response alone is unrealistic. Effective treatment therefore requires a dual-mechanism therapeutic approach. In viable, well-perfused tissues, pathogen control is achieved through targeted systemic antibiotic therapy administered at concentrations corresponding to the minimal inhibitory concentration (MIC). However, in the presence of mature biofilm or highly resistant microorganisms, MIC level exposure is frequently insufficient. In such cases, substantially higher antimicrobial concentrations are required, ideally using agents with demonstrated antibiofilm activity, defined as the minimal biofilm eradication concentration (MBEC) [22,23].

Achieving MBEC-level drug concentrations within the biofilm matrix and across an avital prosthetic interface presents a significant pharmacokinetic challenge. Systemic administration at doses sufficient to reach MBEC would be associated with unacceptable systemic toxicity. Consequently, localized delivery of antimicrobial agents after surgical debridement—either antibiotics, antiseptics, or their combination—represents the most rational and biologically sound strategy for effective biofilm control. This principle of local antimicrobial therapy is well established historically, most notably through the Dakin–Carrel technique introduced during World War I, which demonstrated the effectiveness of high-concentration local antiseptic application in the management of contaminated wounds [33].

Over the past decades, an increasing number of studies have reported favorable outcomes not only with intraoperative antibiotic administration but also with postoperative local antibiotic delivery maintained over a defined treatment period. These techniques have demonstrated success even in infections caused by highly resistant bacterial strains [11,12,27,34,35,36].

In contrast, the evidence supporting the local use of antiseptic agents remains limited. Although their application is theoretically attractive due to broad-spectrum antibacterial activity, potential antibiofilm effects, and antifungal properties, robust clinical studies confirming their efficacy in practical settings are still lacking [18,19,21,22,23,35]. While the search for an ideal antiseptic solution is still ongoing, our findings suggest that a low-cytotoxic formulation based on hypochlorous acid and sodium hypochlorite (Granudacyn^®^, Mölnlycke, Gothenburg, Sweden), used in combination with locally administered antibiotics and incorporated into a structured intra- and postoperative treatment protocol, was feasible and appeared safe in this high-risk cohort. However, the specific contribution of postoperative irrigation or other individual protocol components to infection eradication cannot be determined from the present study design. Although diluted povidone–iodine solutions are more commonly described in arthroplasty literature, our protocol intentionally incorporated brief application of undiluted 10% povidone–iodine as part of a mechanically assisted “microdebridement” strategy. We acknowledge the potential cytotoxicity of higher-concentration povidone–iodine; however, the rationale for this approach was to achieve localized antimicrobial reduction while potentially avoiding more extensive radical soft-tissue excision. This concept remains theoretical and requires further experimental and clinical validation. Importantly, several aspects of the proposed ‘microdebridement’ concept, including antiseptic-guided assessment of tissue surfaces and the potential reduction in synovectomy extent, remain experience-based intraoperative observations rather than validated surgical principles.

### 4.4. Patient Selection and Risk Stratification

The role of host-related factors in the development of periprosthetic joint infection has gained increasing attention in recent years and is now the subject of extensive investigation. Impaired immune status associated with multiple comorbidities may explain the earlier onset of clinical symptoms observed in our patient cohort [8,28]. The fact that pathogenic or opportunistic microorganisms manifest clinically at an earlier stage in immunocompromised hosts has two important implications. On the one hand, it allows for earlier therapeutic intervention, potentially before full biofilm maturation. On the other hand, this may indicate that such patients require more intensive support to achieve effective infection eradication. Therefore, in these patients, the classical DAIR protocol consisting of a single surgical intervention combined with prolonged parenteral antibiotic therapy of 3–6 months may be insufficient. In this context, adjunctive local antimicrobial and antiseptic agents may represent the missing key factor in improving treatment outcomes [4,5,15,37].

Another key factor influencing treatment success is time, defined both as the interval in days or weeks from the index surgical procedure to symptom onset, as well as the duration of symptoms following infection manifestation. Both parameters remain subjects of ongoing debate in the literature. According to current evidence, the most favorable outcomes are achieved when DAIR is performed within four weeks of the index operation and within three weeks of symptom occurrence. Although infection eradication using DAIR beyond these time frames is still possible, both the surgeon and the patient must be aware that success rates are substantially reduced [1,28].

Pathogen virulence represents a critical determinant of treatment strategy and outcome in periprosthetic joint infection. High-virulence pathogens, including Staphylococcus aureus, Gram-negative bacteria, and antimicrobial-resistant strains, typically present with acute and pronounced clinical symptoms, which may facilitate earlier diagnosis and intervention. Despite this, their aggressive tissue invasion, rapid biofilm formation, increased tolerance to antimicrobial agents, and higher propensity for systemic dissemination are associated with elevated failure rates following DAIR [26,30].

Patients undergoing arthroplasty in the setting of acute fracture represent a distinct and particularly vulnerable subgroup with an inherently increased risk for periprosthetic joint infection. These patients frequently exhibit a substantial burden of comorbidities, including cardiovascular disease, diabetes mellitus, renal insufficiency, malnutrition, and immunosuppression, all of which adversely affect host defense mechanisms and wound healing capacity. The cumulative impact of these host-related factors significantly influences both susceptibility to infection and response to treatment, leading some authors to consider fracture-related arthroplasty a relative contraindication to DAIR [2,13,14].

### 4.5. Limitations

This study has several limitations that should be considered when interpreting the results.

First, the retrospective design and small sample size limit the ability to draw definitive conclusions or generalize the findings. However, this study was designed as a feasibility and hypothesis-generating analysis in a consecutive high-risk patient cohort treated with a strictly standardized protocol. The cohort includes only patients treated with the Mursa protocol and does not represent all PJI cases managed at our institution, which may introduce selection bias. If validated in a larger prospective cohort study, our protocol could expand the indications for implant retention strategies in patients at higher risk of failure.

Second, this was a single-center study, and the results may therefore reflect institution-specific surgical expertise, microbiological practices, and perioperative care pathways, which may limit generalizability.

Third, the present cohort represents consecutive patients treated with the Mursa protocol by the developing surgical team rather than all PJI cases managed institutionally during the study period. Treatment strategy selection was individualized according to established surgical principles, including infection chronicity, implant stability, soft-tissue condition, patient-related factors, and overall surgical judgment. Consequently, the possibility of selection bias must be acknowledged, particularly regarding which patients were considered suitable for implant-retention treatment strategies. In addition, because this study was not designed as a registry-based institutional cohort analysis, complete retrospective screening data for all early PJI cases treated during the study period were not reliably available, limiting the ability to construct a formal patient flow analysis.

Fourth, the study lacks a control group treated with conventional DAIR without postoperative local irrigation or sequential antiseptic debridement. Furthermore, because all protocol components were applied simultaneously and no comparator group was available, the relative contribution of individual treatment elements—including antiseptic microdebridement, postoperative irrigation, and selective local antibiotic therapy—could not be isolated. Therefore, no conclusions regarding the incremental therapeutic effect of any individual protocol component can be definitively drawn from the present study. In addition, the specific postoperative irrigation and drainage configuration used in this study has not been independently validated in experimental or comparative clinical studies. Although no irrigation-related complications were observed in this cohort, the safety and incremental effectiveness of this component require formal prospective evaluation.

Fifth, several aspects of the proposed “microdebridement” concept—including antiseptic-guided assessment of tissue surfaces and the potential reduction in synovectomy extent—remain experience-based intraoperative observations rather than validated surgical principles. These concepts should therefore be interpreted cautiously and require further experimental and clinical validation.

Sixth, although follow-up was sufficient to capture early and intermediate failures, long-term outcomes beyond the defined follow-up period remain unknown, and late hematogenous reinfection cannot be excluded. In addition, standardized functional outcome measures and patient-reported outcome scores were not prospectively collected, limiting assessment of postoperative hip function and quality-of-life outcomes.

Finally, antimicrobial regimens were tailored according to pathogen identification and susceptibility, reflecting real-world clinical practice, but introducing potential treatment heterogeneity and confounding by indication. Despite these limitations, the present study provides detailed methodological insight and clinically relevant outcome data in a high-risk patient population often underrepresented in DAIR series.

### 4.6. Clinical Implications and Future Directions

Despite these limitations, the present study provides preliminary evidence that a structured, multimodal DAIR-based approach incorporating local antimicrobial strategies can be implemented safely in patients with early hip PJI, including those with complex clinical profiles.

Future research should focus on prospective, multicenter studies to validate these findings and improve generalizability. Comparative studies evaluating standard DAIR versus DAIR augmented with structured local antimicrobial irrigation are needed to clarify the incremental benefit of this approach. In addition, further work should aim to refine patient selection criteria, define optimal antiseptic agents and exposure parameters, and assess long-term outcomes, including reinfection rates and functional recovery. Such efforts may help establish standardized protocols and better integrate local antimicrobial strategies into contemporary PJI management algorithms.

## Figures and Tables

**Table 1:** Baseline characteristics of the study cohort.

Parameter	Value
Number of patients	16
Sex	10 female/6 male
Mean age (range), years	66.1 (37–91)
ASA classification	
- ASA II	3
- ASA III	13
Charlson Comorbidity Index (CCI), median (range)	5 (0–10)
Comorbidity-polypharmacy score (CPS), median (range)	10.5 (0–25)

## Data Availability

The data supporting the findings of this study are available from the corresponding author upon reasonable request. The data are not publicly available due to ethical restrictions and the protection of patient privacy.

## Abbreviations

The following abbreviations are used in this manuscript:

DAIR	Debridement, Antibiotics, and Implant Retention
PJI	Periprosthetic Joint Infection
EBJIS	European Bone and Joint Infection Society
THA	Total Hip Arthroplasty
ASA	American Society of Anesthesiologists (Physical Status Classification)
CCI	Charlson Comorbidity Index
CPS	Comorbidity–Polypharmacy Score
CRP	C-Reactive Protein
ESR	Erythrocyte Sedimentation Rate
CBC	Complete Blood Count
KLICC	Kidney Disease, Liver Disease, Revision Surgery, Cemented Prosthesis, C-Reactive Protein
MRSE	Methicillin-Resistant Staphylococcus epidermidis
MR-CoNS	Methicillin-Resistant Coagulase-Negative Staphylococci
AVN	Avascular Necrosis
ORIF	Open Reduction and Internal Fixation
MIC	Minimal Inhibitory Concentration
MBEC	Minimal Biofilm Eradication Concentration
